# A Pediatric Case of Urinary Retention Caused by Shingles on the Buttocks

**DOI:** 10.7759/cureus.112547

**Published:** 2026-07-12

**Authors:** Ryutaro Yamada, Tatsuo Fuchigami, Yuko Moriuchi

**Affiliations:** 1 Pediatrics, IMS (Itabashi Medical System) Fujimi General Hospital, Fujimi, JPN; 2 Pediatrics, Nihon University, Tokyo, JPN

**Keywords:** buttocks, chickenpox, elsberg syndrome, flat rash, herpes zoster, papules, shingles, shingles-related urinary retention, urinary retention, valacyclovir

## Abstract

Herpes zoster, also called shingles, is caused by the varicella zoster virus (VZV), which can become active again after lying dormant in nerve cells following chickenpox. Cases of herpes zoster affecting the buttocks in children that lead to urinary retention are rare. This report details a notable case of a 10-year-old girl who experienced urinary retention for nine days before she was hospitalized. Her medical history included chickenpox at approximately one year of age. Five days before the presentation, she visited a clinic where she was advised to undergo urinalysis and was prescribed antibiotics, but her condition did not improve. Around that time, the patient developed a rash on her buttocks and decided to monitor it without further treatment. As her symptoms worsened, she returned for a second urinalysis and was administered antibiotics again without relief. Upon admission, she developed a flat rash near her right buttock, raising suspicion of herpes zoster. Along with urinary issues, she complained of lower abdominal pain, and ultrasonography showed that approximately 800 mL of urine was retained in her bladder. A urinary catheter was inserted, and the patient was treated with valacyclovir. By day 4, the catheter was removed, and the shingles lesions began to crust. The patient was discharged on day 7 with no recurrence. Shingles-related urinary retention in children is extremely rare, making diagnosis challenging owing to the diverse symptoms and rash locations. This case highlights the importance of awareness of such presentations.

## Introduction

Varicella-zoster virus (VZV), which is similar to herpes simplex virus (HSV), is a human herpesvirus that primarily infects nerve cells. It remains dormant in the cranial nerves, dorsal root ganglia of the spinal cord, and ganglia and nerve cells of the autonomic nervous system. When the virus reactivates, often owing to factors such as aging, stress, or immune suppression, it travels along the sensory nerves, causing clusters of papules and small vesicles typical of shingles [1]. Although shingles is common in adults, especially in older adults, it is relatively rare in children. The disease usually affects the chest, and cases involving the sacral region, such as the buttocks, are uncommon [2].

This report describes the case of a child who developed shingles on the buttocks, which led to urinary retention. Elsberg syndrome (ES) is characterized by viral infections, including HSV and VZV, which cause urinary dysfunction and retention. ES is frequently underdiagnosed due to the limited number of reported cases [3]. This pediatric case highlights VZV-related neurological complications and ES.

## Case presentation

A previously healthy 10-year-old Japanese girl presented with difficulty in urinating. Nine days before admission, she noticed that although she felt an urge to urinate, she had trouble passing urine. Five days earlier, she visited a local clinic where a urine test showed leukocyturia. Cefaclor was prescribed for suspected cystitis. Around the same time, the patient developed a rash on her buttocks. Sometimes, she was able to urinate normally; however, at other times, she felt the urge but could barely pass urine. The day before her admission, she underwent another urine test at her previous clinic, which again showed leukocyturia, and she was prescribed cefdinir. However, her condition did not improve, and she was admitted to our hospital on the 10th day of the onset of symptoms. The patient’s medical history included chickenpox at around the age of one year, with no record of vaccination.

**Figure 1:**
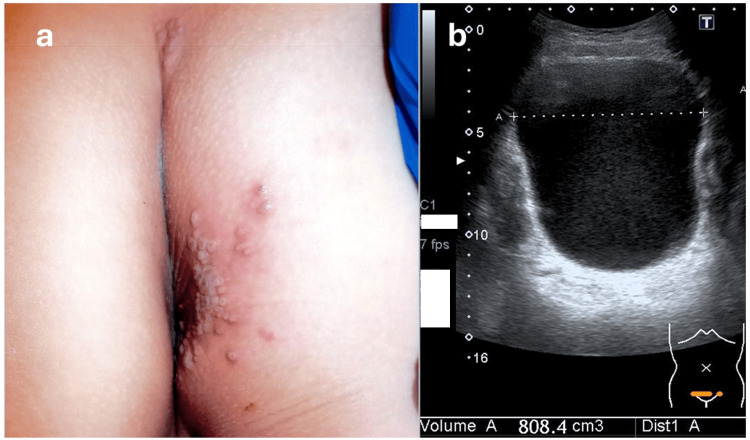
Clinical photograph of the buttocks and ultrasound image of bladder (a) Clusters of blisters observed on the upper and right sides of the intergluteal cleft; (b) The bladder capacity before catheterization was 808 mL, and no obvious irregularities, wall thickening, or neoplastic lesions were observed. No morphological abnormalities were observed in the renal calyces or other parts of the kidney.

Upon her admission to our hospital, her height was 147.5 cm (average height of a Japanese child her age, 146.4 cm), and her weight was 42.1 kg (average weight of a Japanese child her age, 41.14 kg). Her vital signs included a temperature of 36.9°C, a pulse rate of 92 beats per minute, and a respiratory rate of 22 breaths per minute. Chest examination revealed clear breath sounds with no heart murmurs. The abdomen showed moderate bowel sounds, and the lower region was soft and tender. The skin showed clustered vesicles on the upper and right sides of the intergluteal cleft (Figure 1a), but there was no pain in these areas.

Laboratory findings at admission (Table 1) showed a white blood cell count of 7,700/μL and a C-reactive protein level of 0.03 mg/dL, indicating no evidence of elevated inflammatory markers. Blood urea nitrogen was 8.6 mg/dL, and creatinine was 0.35 mg/dL, both within normal ranges, suggesting normal kidney function. Urinalysis was negative for both qualitative proteins and occult blood, with no abnormalities in the red or white blood cell counts of the sediment. A swab taken from the rash site was sent to an outside laboratory for VZV antigen testing using immunochromatography.

**Table 1:** Laboratory Data Blood tests showed no elevated inflammatory markers, and no kidney function abnormalities were observed. Urine tests also showed no significant abnormalities. A swab of the rash revealed a positive result for VZV antigen. ALT: alanine aminotransferase, AST: aspartate aminotransferase, BUN: blood urea nitrogen, Ca: calcium, Cl: chlorine, CRP: C-reactive protein, HPF: high-power field, K: potassium, LDH: lactate dehydrogenase, Na: sodium, P: Inorganic phosphorus, RBC: red blood cell, VZV: varicella zoster virus, WBC: white blood cell.

Test	Result	Reference	Unit	
Blood test				
WBC	7,700	3,300-8,600		/µL
Hemoglobin	13.2	11.6-14.8		g/dL
Hematocrit	41.6	35.1-44.4		%
Platelet	258	158–348		x10^3^/µL
AST	17	8-48		U/L
ALT	9	7–23		U/L
LDH	174	124-222		U/L
Creatinine	0.35	0.46-0.79		mg/dL
Total protein	7.4	6.6-8.1		g/dL
Albumin	4.1	3.8-5.3		g/dL
Na	140	138-145		mmol/L
K	4.0	3.6-4.8		mmol/L
Cl	105	101-108		mmol/L
Ca	9.5	8.8-10.1		mg/dL
P	4.1	2.7–4.6		mg/dL
CRP	0.03	0.01-0.14		mg/dL
Urine test				
Specific gravity	1.016	1.002-1.030		
Urine protein	(–)	(–)		
Urine glucose	(–)	(–)		
Urine occult blood	(–)	(–)		
Sediment RBC WBC	1–4	< 5		/HPF
	1–4	< 5		/HPF
Antigen test for rash				
VZV (immunochromatography)	Positive	Negative		

Abdominal ultrasonography (Figure 1b) revealed a precatheterization bladder capacity of 808 mL. No obvious irregularities, wall thickening, or neoplastic lesions were observed in the bladder, and there were no structural abnormalities in the kidneys, including the renal calyces.

**Figure 2:**
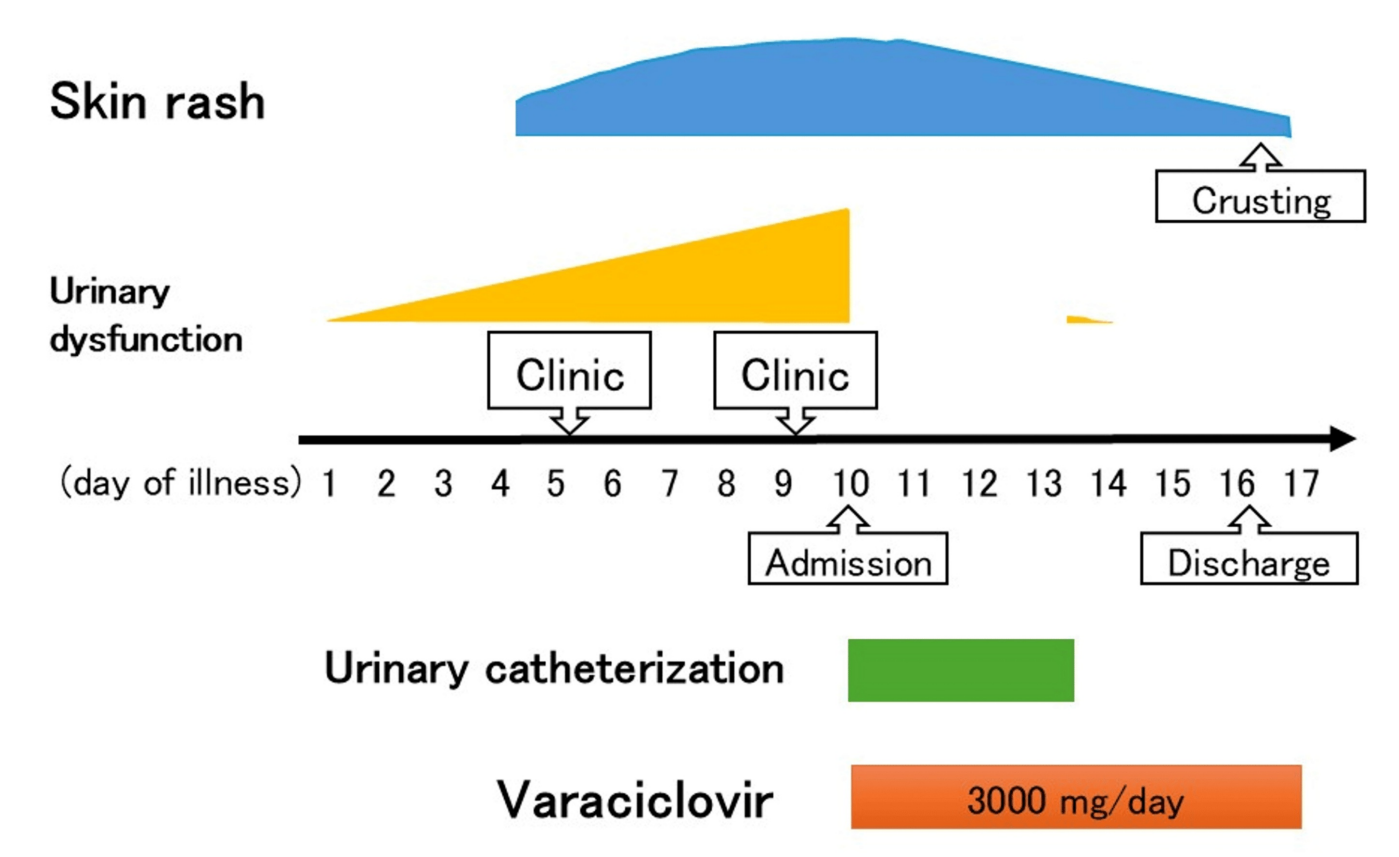
Clinical course Image created with Microsoft Power Point (Microsoft Corporation, Redmond, Washington, United States)

At admission, the patient had experienced significant difficulty in urinating, and due to abdominal distension, a urinary catheter was inserted, draining 1,022 mL of urine. After consulting the dermatology department, the patient was diagnosed with herpes zoster, and treatment with oral valacyclovir hydrochloride (3000 mg/day) was initiated (Figure 2). The rash began to exhibit signs of crusting. The catheter was temporarily removed on the Day 4 of hospitalization (13th day of illness), and she was able to urinate normally after its removal. Although the patient still reported a sensation of residual urine, further abdominal ultrasonography confirmed that none was present. The patient was discharged on Day 7 of hospitalization (16th day of illness). No significant bowel dysfunction was observed during the patient’s hospital stay. The rash healed without pain, and scabs formed. Even 11 days after discharge (20th day of illness), there were no signs of symptom recurrence, and post-void ultrasound showed no residual urine.

## Discussion

This was a rare case of urinary retention caused by herpes zoster in the buttocks of a pediatric patient, highlighting the importance of early diagnosis for better patient outcomes.

Herpes zoster is rare in children, and incidences outside the thoracic area are even rarer. Although herpes zoster is believed to mainly affect people aged 50 years and older, a report by Kim et al. showed an overall incidence rate of 10.4 cases per 1,000 person-years, with only two cases per 1,000 person-years in children, highlighting age-related differences [1]. The thoracic spinal cord region accounts for the highest percentage of cases at 52%. The incidence in the trigeminal nerve area is 12% and increases with age, while the cervical spinal cord, lumbar, and sacral regions account for incidences of 17%, 10%, and 5%, respectively, indicating low occurrence in these areas [2].

Urinary retention is a rare complication of herpes zoster in children. Kang et al. reported that the rate of herpes zoster-related complications in children was 9.0%, with the most common involving the eyes and ears, followed by head and neck rashes, postherpetic neuralgia, bacterial skin infections, and meningitis. Urinary dysfunction was not observed among these complications [4]. Regarding the pathophysiology of VZV-induced neuropathy, the virus can spread to two to three spinal ganglia or extend beyond the anterior horn of the spinal cord to the opposite side. Magnetic resonance imaging (MRI) findings of herpes zoster-related neurological complications, as documented by Shoji et al., revealed patterns of VZV spread, including ascending spinal cord and nerve root involvement, multifocal nerve root involvement, and intraspinal diffusion [5]. In the present case, the skin segments affected by herpes zoster were S2-S4, which contain sensory fibers responsible for bladder filling and the sacral parasympathetic nervous system that controls urination. Damage to this neural region can lead to urinary urgency and dysfunction; however, this also suggests that inflammation extends from the dorsal horn to the lateral medulla and anterior horn, with bilateral impairment likely necessary for urinary retention. We did not fully assess bowel dysfunction or sensory deficits in the affected neural regions. Bilateral involvement is necessary for the development of urinary retention, as observed in this case. Assuming sequential progression from urinary dysfunction to retention, the condition may have evolved from neuritis to myelitis or meningitis. Interestingly, urinary dysfunction was observed before the rash appeared, complicating early diagnosis, as it typically occurs simultaneously with or shortly after the rash begins. Other reports have documented instances in which urinary dysfunction occurred before the appearance of a rash [6].

Among acquired neurogenic bladder syndromes caused by myelitis or meningitis, ES is a condition in which viral infections, including HSV and VZV, lead to urinary dysfunction and retention. ES is often under-recognized because of the limited number of reported cases [3]. First described in a 1913 pathological report by Elsberg and Kennedy [7], ES was documented in five patients presenting with motor, sensory, and autonomic symptoms, without meningitis. It is an infectious syndrome resulting in acute or subacute bilateral lumbosacral radiculitis and lower spinal cord inflammation. Although many reports suggest that ES is primarily triggered by HSV-2, cases involving VZV, cytomegalovirus, Epstein-Barr virus, human immunodeficiency virus, and novel coronavirus have also been reported [3,8]. Some studies have indicated that HSV-2 and VZV together account for 74% of cases [9].

The key differential diagnoses include meningitis-urinary retention syndrome (MRS), Guillain-Barré syndrome (GBS), and acute disseminated encephalomyelitis (ADEM). Cerebrospinal fluid (CSF) analysis, imaging studies, and nerve conduction velocity tests are essential to distinguish between these conditions. Notable differences include meningeal irritation signs and CSF cell counts in MRS, protein-cell dissociation in GBS, and brain imaging findings in ADEM [3,9]. While the definition of ES is not entirely consistent, Savoldi et al. proposed diagnostic criteria (Table 2) based on clinical records from the Mayo Clinic and existing literature [8]. In this case, despite several limitations in the clinical examination and testing for differential diagnosis, the presence of herpes zoster and acute or subacute urinary retention led us to classify it as “clinically probable and strongly suggestive of ES.”

**Table 2:** Elsberg syndrome according to diagnostic certainty ^A. Required^ ^A1. The clinical symptoms and signs of cauda equina involvement include urinary hesitancy or retention, bowel incontinence, or severe constipation (erectile dysfunction insufficient on its own).^ ^A2. MRI or electrophysiological evidence of cauda equina involvement: enhancement of the cauda equina; EMG evidence of radiculopathy.^ ^B. Supportive but not required^ ^B1. Time course: acute/subacute onset, no relapse, progression for less than 3 months^ ^B2. Coexisting or recently preceding symptoms of genital herpes infection or other clinical symptoms of herpes virus infection.^ ^B3. Clinical (e.g., exaggerated reflexes and Babinski signs) or MRI evidence of myelitis in the conus.^ ^B4. CSF pleocytosis^ ^B5. Documented herpes virus infection in CSF by PCR, culture, or detection of IgM serology^ ^C. Red flags^ ^C1. Relapses beyond 1 y from onset^ ^D. Exclusionary^ ^D1. Myelitis extending rostrally to T9^ ^D2. Other neurological symptoms suggestive of an alternative etiology: optic neuritis and brain/brainstem syndrome.^ ^D3. Other etiologies proven/more likely for syndrome: NMOSD, dural arteriovenous fistula, viral transverse myelitis, and other causes of myelopathy^ CSF: cerebrospinal fluid, EMG: electromyogram, IgM: immunoglobulin M, MRI: magnetic resonance imaging, NMOSD: neuromyelitis optica spectrum disorder, PCR: polymerase chain reaction Source: Savoldi et al., 2017 [8]; available under the Creative Commons CC-BY-NC-ND

Categories	Criteria
Laboratory-supported definite	(A1 or A2) and B5
Clinically definite	A1 or A2; B1 and two of B2–B4; B1 and B2 (if concomitant)
Clinically probable	A1 or A2; B1 and one of B2–B4
Clinically possible	A1 or A2; one of B1–B4
Excluded	Neither of A1 nor of A2; any of D1–D3

The treatment of urinary dysfunction and retention caused by herpes zoster generally involves standard therapies, such as antiviral drugs and steroids, along with conservative measures such as catheterization [9]. Evidence has shown that antiviral therapy can improve the prognosis of patients with herpes zoster-related complications. Although antiviral treatments have been proven to enhance outcomes, the effectiveness of immunotherapies such as pulse steroid therapy remains unconfirmed [9].

The recovery time can range from several days to several months, with an average of two to three weeks [6,10]. In the current case, urinary dysfunction lasted for approximately two to three weeks. Although it is unclear whether antiviral therapy shortened the duration of urinary retention, the symptoms worsened until treatment was initiated. Therefore, early administration of antiviral medications may slow disease progression. Qadri et al. recommended initiating these therapies when clinical signs suggested ES, even if laboratory results were pending, owing to the risk of ascending myelitis [9]. Furthermore, in the current case, significant urinary retention was observed upon admission, making early intervention crucial for preventing the complications and sequelae of bladder overdistension.

Urinary dysfunction at the sacral level due to herpes zoster-related nerve palsy is often underdiagnosed in adults [6]. The rash most frequently associated with urinary dysfunction appears in the S2-S4 region; however, this area has a low incidence, making examination difficult in children. It is well known that shingles pain tends to be less severe in children, partly because postherpetic neuralgia is less common among them. Additionally, because of their age, pediatric patients may have vague symptoms, and examining the buttocks or perianal area can be challenging due to cooperation issues. As early antiviral treatment can improve outcomes for this disease [9], it is important to check for rashes on the buttocks or lower limbs or assess any sensory discomfort in these areas when urinary dysfunction occurs, even if no rash is visible. This approach aids in early diagnosis and intervention. In this case, there were no signs of impaired consciousness, headache, vomiting, or other neurological abnormalities; thus, more invasive tests, such as CSF analysis, intracranial imaging, and nerve conduction studies, were not performed. Additionally, there have been no quantitative assessments of urinary dysfunction. Although the main symptoms were rash and urinary retention, bowel function, also linked to the same nerve area, was not evaluated, nor was sensation tested outside the rash area. In cases that progress to urinary retention, CSF analysis and imaging are necessary for differential diagnosis. Collecting findings related to this nerve region will be valuable in future clinical practice.

## Conclusions

This was a pediatric case of urinary retention following herpes zoster infection. Although children often find it difficult to clearly express their symptoms, herpes virus infections such as VZV in the lumbosacral region should be included in the differential diagnosis of acquired urinary dysfunction. Early diagnosis and treatment are essential to prevent worsening of the condition. Additionally, because such cases are rare in children, more data, including diagnostic test results, should be collected to enhance our understanding.
